# Chemotactic behavior of egg mitochondria in response to sperm fusion in mice

**DOI:** 10.1016/j.heliyon.2018.e00944

**Published:** 2018-11-16

**Authors:** Maki Iwai, Yuichirou Harada, Rinako Miyabayashi, Woojin Kang, Akihiro Nakamura, Natsuko Kawano, Yoshitaka Miyamoto, Mitsutoshi Yamada, Toshio Hamatani, Mami Miyado, Keiichi Yoshida, Hidekazu Saito, Mamoru Tanaka, Akihiro Umezawa, Kenji Miyado

**Affiliations:** a Department of Obstetrics and Gynecology, Keio University School of Medicine, 35 Shinanomachi, Shinjuku, Tokyo 160-8582, Japan; b Department of Reproductive Biology, National Research Institute for Child Health and Development, 2-10-1 Okura, Setagaya, Tokyo 157-8535, Japan; c Department of Molecular Pathology, Tokyo Medical University, 6-1-1 Shinjuku, Shinjuku, Tokyo 192-0397, Japan; d Shinbashi YUME Clinic, 2-5-1 Shinbashi, Miyato, Tokyo 105-0004, Japan; e Department of Perinatal Medicine and Oocyte Care, National Center for Child Health and Development, 2-10-1 Okura, Setagaya, Tokyo 157-8535, Japan; f Department of Life Sciences, School of Agriculture, Meiji University, 1-1-1 Higashimita, Tama, Kawasaki, Kanagawa 214-8571, Japan; g Department of Molecular Endocrinology, National Research Institute for Child Health and Development, 2-10-1 Okura, Setagaya, Tokyo 157-8535, Japan; h Advanced Medicine, Innovation and Clinical Research Center, Tottori University Hospital, 36-1 Nishicho, Yonago, Tottori 683-8504, Japan

**Keywords:** Cell biology, Developmental biology, Molecular biology, Zoology

## Abstract

Mitochondria are the powerhouses of eukaryotic cells and their positioning contributes to fertilization and early developmental processes. We report that sperm fusion triggers Ca^2+^ oscillations and mitochondrial movement toward fused sperm (mitochondrial chemotaxis) in mouse eggs. Mitochondria functioned in Ca^2+^ storage and were colocalized with endoplasmic reticulum (ER) during Ca^2+^ oscillations. Mitochondria then moved toward the fused sperm. Sperm extracts lacking nuclei induced Ca^2+^ oscillations, but did not promote mitochondrial chemotaxis. Our results suggest that sperm fusion motivates Ca^2+^ oscillation-independent mitochondrial chemotaxis. This phenomenon indicates that egg mitochondria interact with sperm materials, presumably nuclear substances, and their network tethers egg and sperm nuclei at the early stage of zygote formation.

## 1 Introduction

Mitochondria are important organelles for ATP production in eukaryotic cells and the regulation of cell functions [1]; accordingly, they are important determinants of the success of fertilization and early embryonic development. They are not autonomous, but form an interconnected network that varies in structure according to cell functions [2]. Mitochondrial replacement has recently emerged as a promising strategy to prevent the transmission of mitochondrial diseases and infertility [3]. Accordingly, understanding mitochondrial dynamics has substantial implications for basic biology and medical science.

Mitochondrial fusion and fission generate mitochondria with distinct morphologies and interconnected networks [4]. In animals, mitochondria are strictly inherited from a single parent, usually the mother. After fertilization, sperm mitochondria are eliminated by diverse mechanisms, including ubiquitination-proteasomal degradation [5], mitophagy [6], and allophagy [7]. However, the biological significance of the uniparental transmission of mitochondria remains mysterious.

When cells are exposed to external stimuli, calcium ions (Ca^2+^) are released from intracellular stores, such as the endoplasmic reticulum (ER), leading to oscillatory rises in the intracellular Ca^2+^ concentration (Ca^2+^ oscillation) or transient rises [8]. Accordingly, Ca^2+^ is categorized as a second messenger involved in transducing external signals to intracellular events [9]. After fertilization, mammalian eggs transition from the metaphase-arrested state of the second meiotic division (MII), termed egg activation, and this is initiated by Ca^2+^ oscillation [10]. In ascidians, sperm-triggered Ca^2+^ oscillations are transduced into mitochondrial Ca^2+^ signals that stimulate mitochondrial respiration [11]. Mitochondria may function to absorb intracellular Ca^2+^ by the concentration of heterogeneously distributed mitochondria in eggs after sperm fusion, leading to Ca^2+^ oscillations. Furthermore, Ca^2+^ oscillations may be coordinated by the interplay between ER and mitochondrial activities in eggs. However, the precise links between Ca^2+^ oscillations, mitochondrial movement, and sperm fusion have not been established.

To study mitochondrial dynamics during Ca^2+^ oscillations, we monitored mouse eggs after sperm fusion using eggs labelled with fluorescent biomarkers.

## 2 Materials and methods

### 2.1 Reagents

A red-fluorescent dye that stains mitochondria in living cells in a membrane potential-dependent manner (MitoTracker Red CMXRos), a visible light-excitable calcium indicator (Oregon Green 488 BAPTA-1, AM), and a photostable probe selectively stains the ER in living cells (ER-Tracker Blue-White DPX) were purchased from Invitrogen Corp (Waltham, MA, USA). Egg and sperm nuclei were counterstained with 4′,6-diamidino-2-phenylindole (DAPI) (Invitrogen Corp.). Trifluoromethoxy carbonylcyanide phenylhydrazone (FCCP), a potent uncoupler of oxidative phosphorylation in mitochondria that disrupts membrane potential and ATP synthesis by transporting protons across cell membranes [12], was purchased from Sigma-Aldrich Co. (St. Louis, MO, USA).

### 2.2 Collection of mouse eggs and sperm

Female C57BL/6N mice (8–12 weeks old; purchased from Japan SLC Inc., Shizuoka, Japan) received intraperitoneal injections of 5 IU of pregnant mare's serum gonadotropin (PMSG; Merck4Biosciences, Darmstadt, Germany) followed by 5 IU of human chorionic gonadotropin (hCG; Merck4Biosciences) 46–48 h apart. Eggs at metaphase II were collected from the oviduct of females 14–16 h after hCG administration. To collect cumulus-removed, zona-intact eggs (zona-intact eggs), cumulus cells were dispersed from eggs by incubation for 10 min at 37 °C in TYH medium or M2 medium containing hyaluronidase (300 μg/ml; Merck4Biosciences). The eggs were then incubated in TYH medium or M2 medium. To collect eggs lacking the zona pellucida (zona-free eggs), ovulated eggs were incubated with collagenase (WAKO#034-10533; Wako Pure Chemical Industries Ltd., Osaka, Japan) at a final concentration of 0.1 mg/ml for 5 min at 37 °C in TYH medium or M2 medium. Sperm collected from the epididymides of 8- to 12-week-old B6C3F1 male mice were capacitated by incubation in TYH medium for 30 min in an atmosphere of 5% CO_2_ in air at 37 °C before insemination.

All mice were housed under specific pathogen-free controlled conditions. Food and water were available ad libitum. The procedures for animal experiments were in accordance with the principles and guidelines of the Institutional Animal Care and Use Committee of the National Research Institute for Child Health and Development and were approved by this committee (No. A2004-004-C13).

### 2.3 Measurement of the Ca^2+^ concentration and mitochondrial membrane potential

Zona-free eggs were incubated in medium containing 500 nM MitoTracker Red CMXRos, 5 μM Oregon Green 488 BAPTA-1, AM, and 5 μg/ml DAPI for 30 min at 37 °C, and were washed twice in TYH medium. The eggs were transferred to a 2-μl drop of TYH medium in glass-based dishes (35 mm; IWAKI Glass Co. Ltd., Tokyo, Japan) and fixed by covering with a square coverslip (18 mm × 18 mm) spotted with white Vaseline. TYH medium was added to the eggs from the slit of coverslips. The sperm (final concentration, 5 × 10^2^ sperm/ml) were then added to the TYH medium containing eggs. The eggs were monitored with CSU-Frontier (Yokogawa Corp., Tokyo, Japan) by incubation in TYH medium in an atmosphere of 5% CO_2_ in air at 37 °C. The serial Z-stack images were reconstructed as 3-dimentional (3D) movies.

Zona-intact eggs were incubated in M2 medium or Ca^2+^-free M2 medium containing 500 nM MitoTracker Red CMXRos and 5 μM Oregon Green 488 BAPTA-1, AM for 30 min at 37 °C, and were washed twice in new media. The eggs were transferred to a glass-based dish (35 mm) and covered with a square coverslip (18 mm × 18 mm) sealed with double-faced tape (NITOMS, Inc., Tokyo, Japan). The eggs were filled with M2 medium or Ca^2+^-free M2 medium. The eggs were monitored using a confocal laser microscope (LSM510Meta; ZEISS, Jena, Germany). After 5 min of incubation, FCCP was added to the medium at a final concentration of 1 μM. After 10 min of incubation, fresh M2 medium or Ca^2+^-free M2 medium was added to eggs and the medium containing FCCP was wiped with a paper towel. The eggs were monitored for an additional 10 min. The serial Z-stack images were reconstructed as 3D movies.

### 2.4 Observation of the ER and mitochondria

Zona-intact eggs were stained with 500 nM MitoTracker Red CMXRos and 1.7 μM ER-Tracker Blue-White DPX for 30 min in TYH medium, and were washed twice in fresh TYH media. After the eggs were washed, they were transferred to a 3-μl drop of TYH medium and monitored using a confocal laser microscope (LSM510Meta). The sperm (final concentration, 5 × 10^2^ sperm/ml) were then added to the medium containing the eggs. After 3 h of insemination, fertilized eggs were observed using a confocal laser microscope (LSM510Meta). The serial Z-stack images were reconstructed as 3D movies.

### 2.5 Ca^2+^ oscillation in Cd9^−/−^ eggs

Zona-free eggs were collected from 8 to 12-week-old female C57BL/6N mice (*Cd9*^+/+^) and *Cd9*^−/−^ mice. As reported previously [13, 14], *Cd9*-deficient eggs are defective in sperm fusion, but sperm adhesion occurs normally. Thus, we determined whether sperm adhesion contributes to mitochondrial chemotaxis by monitoring zona-free *Cd9*-deficient eggs adhered to sperm in comparison with wild-type eggs adhered and fused to sperm. They were incubated in M2 medium containing 5 μM Oregon Green 488 BAPTA-1, AM and 5 μg/ml DAPI for 30 min, and were washed twice for 10 min in fresh M2 medium. After the eggs were washed, they were transferred to glass-based dishes (35 mm), and fixed by covering with a square coverslip (18 mm × 18 mm) spotted with white Vaseline. The 15-μl TYH medium was added to the eggs from the slit of coverslips. The sperm (final concentration, 5 × 10^2^ sperm/ml) were then added to the medium containing the eggs. Then, the eggs were monitored using CSU-Frontier (Yokogawa Corp.) by incubation in M2 medium in an atmosphere of 5% CO_2_ in air at 37 °C. The serial Z-stack images were reconstructed as 3D movies.

### 2.6 Microinjection of mouse sperm extracts

Sperm were collected from the epididymides of 8- to 12-week-old B6C3F1 male mice and washed 3 times in phosphate-buffered saline. After the sperm were washed, they were suspended in intracellular-like medium (ICM) containing 120 mM KCl, 20 mM HEPES, 0.1 nM EGTA, 10 mM Na-β-glycerophosphate, 0.2 mM PMSF, and 1 mM DTT. The sperm suspension was treated 5 times by ultrasonication for 20 s and centrifuged for 30 min at 4 °C at 12,000 × *g*. The supernatants (mouse sperm extracts) were frozen at −80 °C. The mSE was injected into the zona-intact eggs in Ca^2+^-free HEPE-CZB medium. The volume of injected mSE was set to 1–5% of the egg volume. The serial Z-stack images were reconstructed as 3D movies. In addition, mSE were resolved by sodium dodecyl sulfate-polyacrylamide gel electrophoresis (SDS-PAGE) on 12% acrylamide gels and stained with Coomassie Brilliant Blue (CBB) and silver stain solutions.

### 2.7 Treatment with cytoskeletal disruptors

Zona-free eggs were stained with 500 nM MitoTracker Red CMXRos, 1.7 μM ER-Tracker Blue-White DPX for 30 min in TYH medium, and were washed twice in fresh TYH media. The eggs were treated with 5 μM nocodazole or 24 mM latrunculin A for 10 min. After treatment, the eggs were transferred to glass-based dishes (35 mm), and fixed by covering with a square coverslip (18 mm × 18 mm) spotted with white vaseline. The 15-μl TYH medium was added to the eggs from the slit of coverslips. The sperm at the final concentration of 5 × 10^2^ sperm/ml were then added to the medium containing the eggs. The eggs were monitored with CSU-Frontier (Yokogawa Corp., Tokyo Japan) by incubating them in M2 medium in an atmosphere of 5% CO_2_ in air at 37 °C. In addition, cumulus-intact eggs were collected from C57BL/6N and treated with 24 mM latrunculin A. After washing, the sperm at the final concentration of 1 × 10^5^ sperm/ml were added to the eggs, and the rate of eggs at the pronuclear stage was estimated by confocal-microscopic observation.

### 2.8 Statistical analysis

Comparisons were evaluated by one-way analysis of variance following by post-hoc Scheffe's tests, Mann–Whitney U-tests, or Fisher's exact tests. Statistical significance was defined as P < 0.05. Results are expressed as means ± standard error of the mean (SEM).

## 3 Results

### 3.1 Ca^2+^ oscillation and mitochondrial membrane potential

**Fig. 1:**
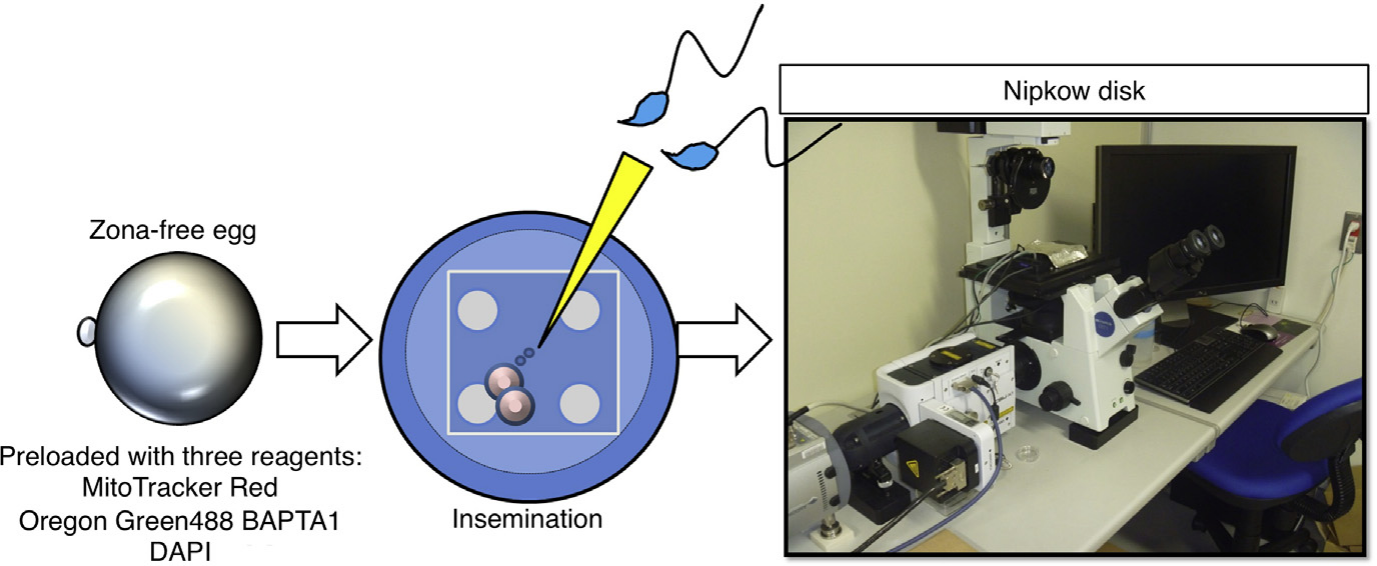
Experimental flow. Zona-free eggs were incubated in the medium containing 500 nM MitoTracker Red CMXRos, 5 μM Oregon Green 488 BAPTA-1, AM, and 5 μg/ml DAPI for 30 min. Eggs were transferred to a 2-μl drop of TYH medium in glass-based dishes (35 mm, IWAKI Glass Co. Ltd., Tokyo, Japan), and fixed by covering with a square coverslip (18 mm × 18 mm) spotted with white Vaseline. The sperm (5 × 10^2^ sperm/ml) were then added to the TYH medium containing the eggs. The eggs were monitored with CSU-Frontier (Yokogawa Corp., Tokyo Japan) by incubation in TYH medium.

We examined the relationship between mitochondrial activity and the intracellular Ca^2+^ concentration after sperm fusion (Fig. 1). Mitochondrial membrane potential is critical for maintaining the physiological function of the respiratory chain to generate ATP [15]. Since the eggs are covered with cumulus cells and the egg coat, i.e., the zona pellucida, the time of onset of sperm fusion cannot be precisely controlled. Therefore, to standardize this time, we used cumulus cell- and zona-free eggs.

**Fig. 2:**
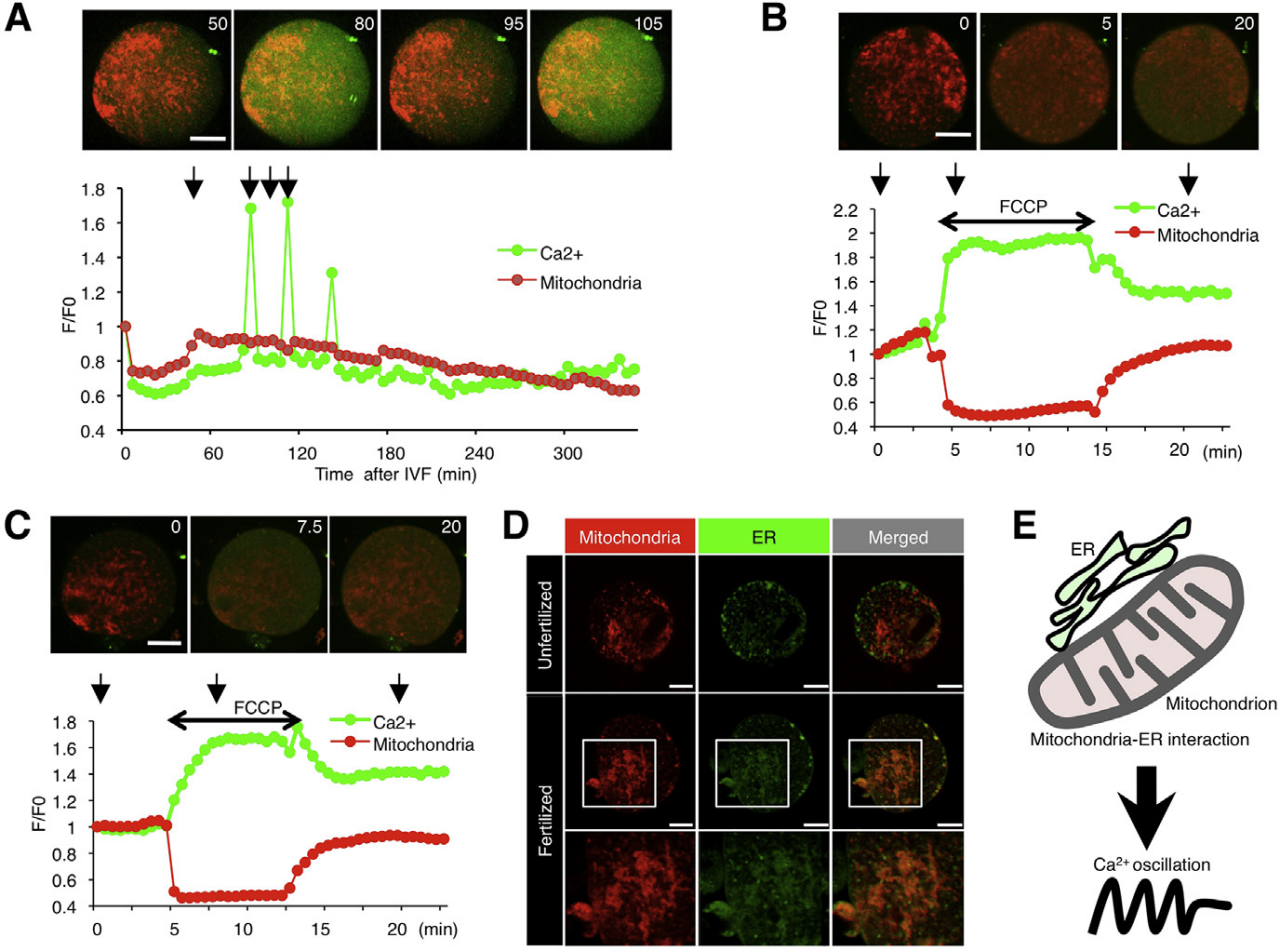
Ca^2+^ oscillation and mitochondrial membrane potential. A, Ca^2+^ oscillation and mitochondrial membrane potential. Images corresponding to arrows in the graph (50, 80, 95, and 105 min after insemination) are shown above. Green line, Ca^2+^ oscillation in a zona-free, sperm-fused egg stained with Oregon Green 488 BAPTA-1, AM; red line, membrane potential of mitochondria labeled with MitoTracker Red CMXRos in a zona-free, sperm-fused egg. Scale bar, 20 μm. B, Effect of FCCP treatment on Ca^2+^ oscillation and mitochondrial membrane potential in M2 medium. A double-headed arrow indicates the period of FCCP treatment. Images corresponding to arrows in the graph (0, 5, and 20 min after monitoring) are shown above. Scale bar, 20 μm. C, Effect of FCCP treatment on Ca^2+^ oscillation and mitochondrial membrane potential in Ca^2+^-free M2 medium. A double-headed arrow indicates the period of FCCP treatment. Images corresponding to arrows in the graph (0, 7.5, and 20 min after monitoring) are shown above. Scale bar, 20 μm. D, Localization of mitochondria and ER in unfertilized and fertilized cumulus-intact eggs. Eggs were stained with MitoTracker Red CMXRos and ER Tracker Blue-White DPX. Upper images, unfertilized egg; middle and lower images, fertilized eggs. Scale bar, 20 μm. E, Schematic model of mitochondria-ER interaction during Ca^2+^ oscillation.

When eggs were monitored from the time of sperm addition (set to 0 min) (Fig. 2A and Movie 1), the first Ca^2+^ rise was recorded at 80 min and repetitive Ca^2+^ rises continued until 140 min. In this experiment, eggs were scanned every 5 min; accordingly, the number of Ca^2+^ rises was less than previous estimates for eggs scanned at 1-min intervals [10, 16]. As reported previously [10, 16], Ca^2+^ was evenly distributed throughout the egg cytoplasm (80 and 105 min in Fig. 2A). Mitochondrial membrane potential increased moderately after sperm addition and returned to the steady state after the end of Ca^2+^ oscillations. This pattern appeared to be correlated to the period of Ca^2+^ oscillations.

### 3.2 Mitochondrial membrane potential and Ca^2+^ oscillation

We examined the relationship between mitochondrial membrane potential and intracellular Ca^2+^ concentration. In particular, we examined unfertilized eggs transiently treated with FCCP. As shown in Fig. 2B and Movie 2, FCCP treatment and removal reduced and increased mitochondrial membrane potential in cumulus-free and zona-intact eggs (zona-intact eggs), corresponding to the increase and reduction of the Ca^2+^ concentration at 5 and 20 min. To restrict Ca^2+^ reservoirs to the egg cytoplasm, we next incubated eggs in Ca^2+^-free M2 medium (Fig. 2C and Movie 3). In the Ca^2+^-free M2 medium, the patterns in FCCP-treated zona-intact eggs were quite similar to those of eggs in conventional M2 medium at 7.5 and 20 min.

### 3.3 Colocalization of mitochondria and ER after sperm fusion

Membrane contact sites between ER and mitochondria are hotspots for Ca^2+^ signaling [17, 18, 19]. To examine the interaction between ER and mitochondrial membranes, we compared their localization in zona-intact eggs before and after sperm fusion. Mitochondria and ER were labeled with MitoTracker Red CMXRos and ER Tracker Blue-White DPX, respectively (Fig. 2D). ER was patchily distributed mainly underneath the plasma membrane, whereas mitochondria were localized mainly in the inner space (upper images in Fig. 2D). The ER colocalized with mitochondria at 3 h after insemination (middle and lower images in Fig. 2D). To perform long-time observation of living eggs without cellular damage, the eggs were scanned every 5 min. Although Ca^2+^ rises were frequently missed under this condition, Ca^2+^ oscillation was predicted to serially occur beyond 300 min (Fig. 2A), because Ca^2+^ oscillation corresponds to mitochondrial activity. Additionally, mitochondria partially colocalized with ER even in unfertilized eggs. Thus, we assumed that ER-mitochondria colocalization might be involved in long-lasting Ca^2+^ oscillation (Fig. 2E).

### 3.4 Mitochondrial distribution before and after Ca^2+^ oscillation

**Fig. 3:**
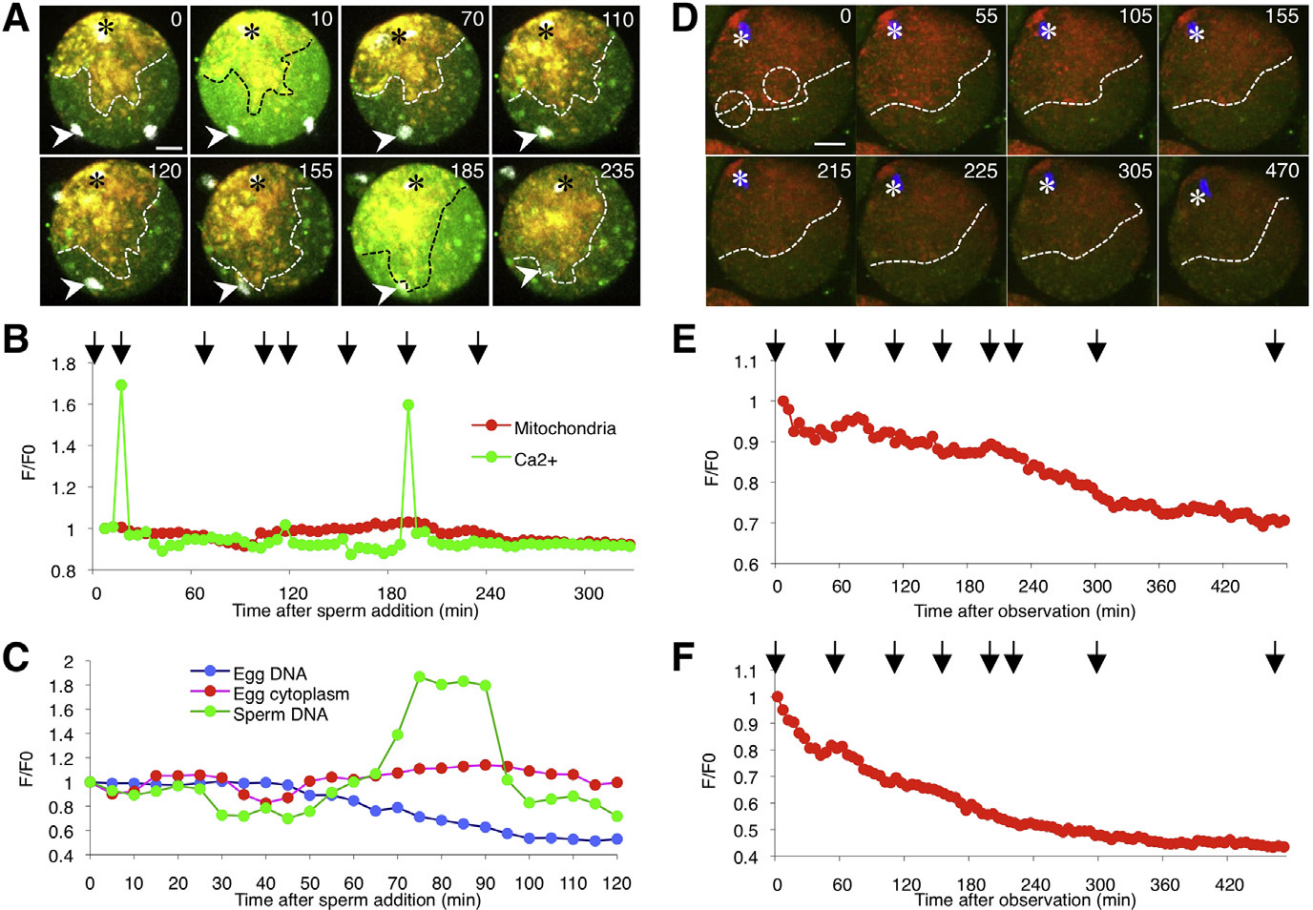
Mitochondrial distribution before and during Ca^2+^ oscillation. A, Mitochondrial distribution in a fertilized egg. The egg was stained with MitoTracker Red CMXRos and Oregon Green 488 BAPTA-1, AM. Images (corresponding to 0, 10, 70, 110, 120, 155, 185, and 235 min after insemination) are shown. Arrowheads indicate the fused sperm heads. Dotted lines, front edge of the mitochondrial distribution. Asterisks, egg nuclei. Scale bar, 20 μm. B, Fluorescence monitored in the whole egg after sperm addition. C, Mitochondrial fluorescence monitored in 3 areas (egg DNA, egg cytoplasm, and sperm DNA). The fluorescence was estimated by the intensity of MitoTracker Red as mitochondria surrounding metaphase-II chromosomes, the sperm nucleus, and in the egg cytoplasm. D, Mitochondrial distribution in an unfertilized egg. The egg was stained with MitoTracker Red CMXRos and Oregon Green 488 BAPTA-1, AM. Images (corresponding to 0, 55, 105, 155, 215, 225, 305, and 470 min after monitoring) are shown. Dotted lines, front edge of the mitochondrial distribution. Dotted circles, monitored areas (E and F). Asterisks, egg nuclei. Scale bar, 20 μm. E, F, Mitochondrial fluorescence monitored in dotted circles of D. Arrows in the graph show the time after monitoring (0, 55, 105, 155, 215, 225, 305, and 470 min).

To examine the mitochondrial distribution during Ca^2+^ oscillation, we monitored zona-free eggs stained with MitoTracker Red CMXRos and Oregon Green 488 BAPTA-1, AM before and after sperm addition (Fig. 3A, B and Movie 4). At 10 min after sperm addition, the sperm fused to the egg plasma membrane (arrowheads in Fig. 3A), and the first Ca^2+^ rise was observed. At 70 min, polar body extrusion began. Mitochondria began to move from 110 min and reached sperm nuclei at 155 min (dotted lines in Fig. 3A). The final Ca^2+^ rise occurred at 185 min after sperm addition. Moreover, fluorescence increased only for sperm DNA, and not for egg DNA or egg cytoplasm, from 65 to 95 min after sperm addition (Fig. 3C). Concurrently, we monitored mitochondria in unfertilized eggs (Fig. 3D and Movie 5). Mitochondria were stationary throughout the monitoring period in the eggs without sperm fusion (dotted lines in Fig. 3D). The fluorescence (dotted circles in Fig. 3D) reduced gradually (Fig. 3E, F).

### 3.5 Stimuli for mitochondrial movement

**Fig. 4:**
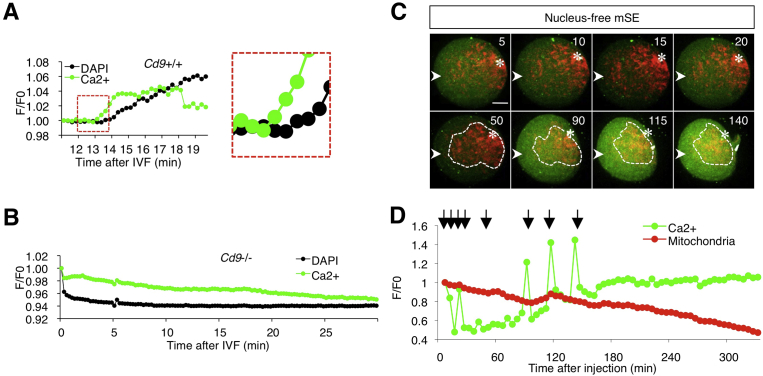
Mitochondrial distribution in the egg after mSE injection. A, Ca^2+^ rise in a wild-type egg after sperm adhesion. A dot-lined box, Ca^2+^ rise from sperm adhesion to fusion. Green line, Ca^2+^ rise; black line, fluorescent intensity of DAPI in the sperm. B, Ca^2+^ rise in a *Cd9*-deficient egg after sperm adhesion. Green line, Ca^2+^ rise; black line, fluorescent intensity of DAPI in the sperm. C, Effect of mouse sperm extracts on mitochondrial redistribution. Images (corresponding to 5, 10, 15, 20, 50, 90, 115, and 140 min after injection) are shown. Mouse sperm extracts was injected into a site shown in a diagram. Dotted areas, accumulated mitochondria. Asterisks, egg nuclei. Scale bar, 20 μm. D, Fluorescence monitored in the whole egg after mSE injection. Arrows in the graph show the time after mSE injection (5, 10, 15, 20, 50, 90, 115, and 140 min).

In non-mammalian vertebrates, sperm adhesion induces a transient Ca^2+^ rise in eggs, leading to egg activation [20]. As reported previously [13, 14], *Cd9*-deficient eggs are defective in sperm fusion, but sperm adhesion occurs normally. Thus, we determined whether sperm adhesion contributes to mitochondrial chemotaxis by monitoring zona-free *Cd9*-deficient eggs adhered to sperm in comparison with wild-type eggs adhered and fused to sperm. Eggs were stained with Oregon Green 488 BAPTA-1, AM. Eggs were preloaded with DAPI, and we were able to easily determine the beginning of sperm fusion by DAPI transfer from eggs to the sperm nuclei [21]. In wild-type eggs, a Ca^2+^ rise occurred before DAPI incorporation into the sperm DNA (box in Fig. 4A). We next monitored the Ca^2+^ concentration in *Cd9*-deficient eggs (Fig. 4B), but no Ca^2+^ rise was detected. This result implies that sperm fusion increases the Ca^2+^ concentration in the egg cytoplasm, leading to mitochondrial chemotaxis.

### 3.6 Insufficient effect of sperm extracts on mitochondrial chemotaxis

To examine the role of sperm materials in mitochondrial chemotaxis, sperm extracts lacking nuclei were injected into zona-intact eggs and mitochondrial chemotaxis was monitored (Fig. 4C and Movie 6). The mouse sperm extracts were injected into the egg in the left region (indicated by arrowheads in the figure). The first Ca^2+^ rise was observed at 5 min after mSE injection. The sperm extracts induced Ca^2+^ oscillations at 10, 20, 90, 115, and 140 min (Fig. 4D), but not mitochondrial chemotaxis (dotted circles in Fig. 4C), and mitochondrial activity was reduced gradually (Fig. 4D).

### 3.7 Components in mouse sperm extracts

**Fig. 5:**
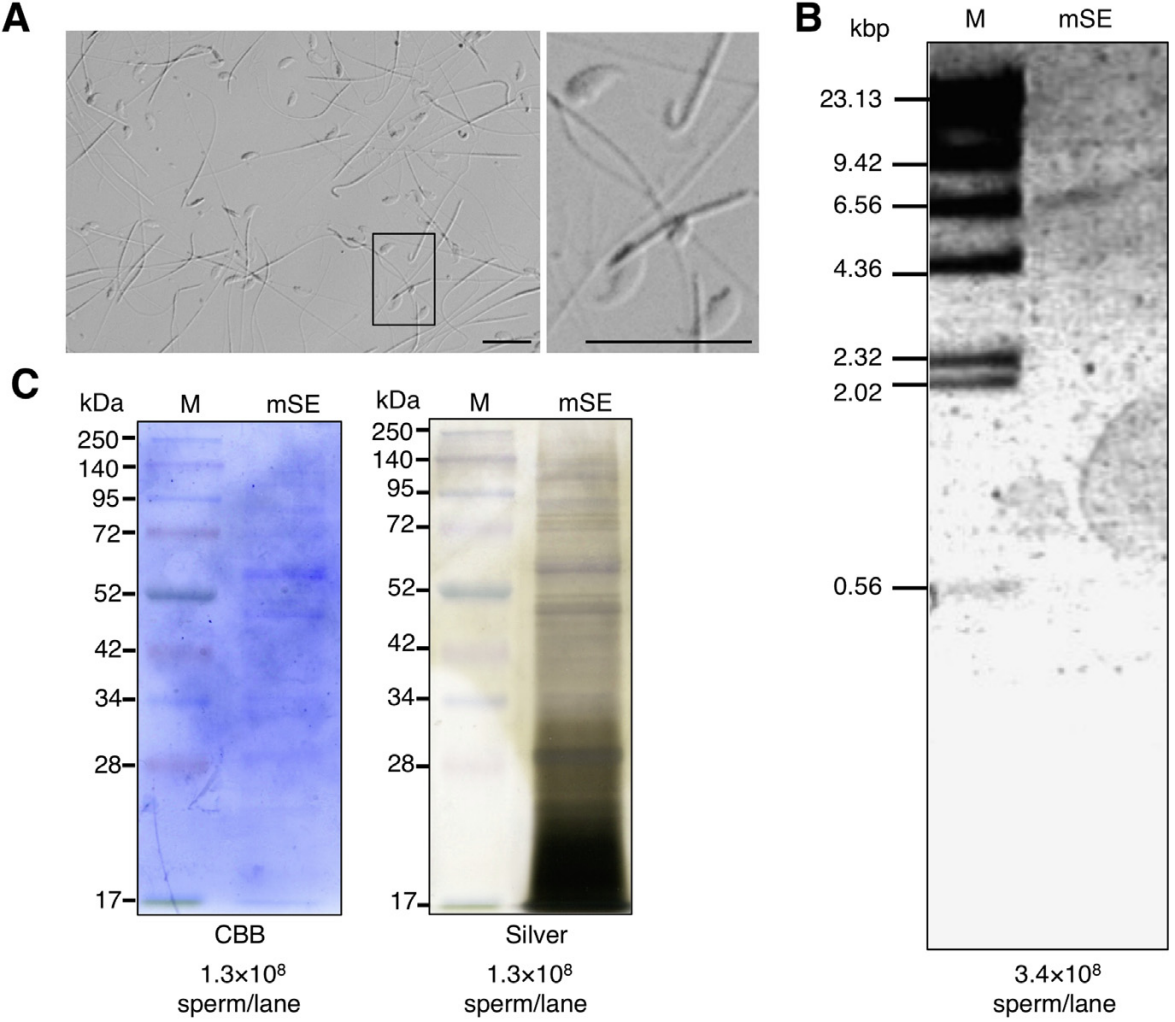
Component analysis of the mSE. A, Microscopic observation of the sperm after ultrasonication. Right panels, enlarged image of the box in the left image. Scale bar, 20 μm. B, Agarose gel electrophoresis of the mSE. C, The SDS-PAGE gel stained with CBB and silver stain solutions.

To explore the components that trigger mitochondrial redistribution, we examined the components included in the mSE after ultrasonication. The sperm heads were sustained microscopically in spite of ultrasonication (Fig. 5A). After centrifugation, nucleotides were below the detectable level (Fig. 5B). Otherwise, multiple proteins were detected in the mSE (Fig. 5C). Form this result, we hypothesize that in the absence of detectable DNA/RNA in the ultrasonicated sperm heads, these molecules contribute to the mitochondrial attraction.

### 3.8 Contribution of cytoskeletons to mitochondrial redistribution

**Fig. 6:**
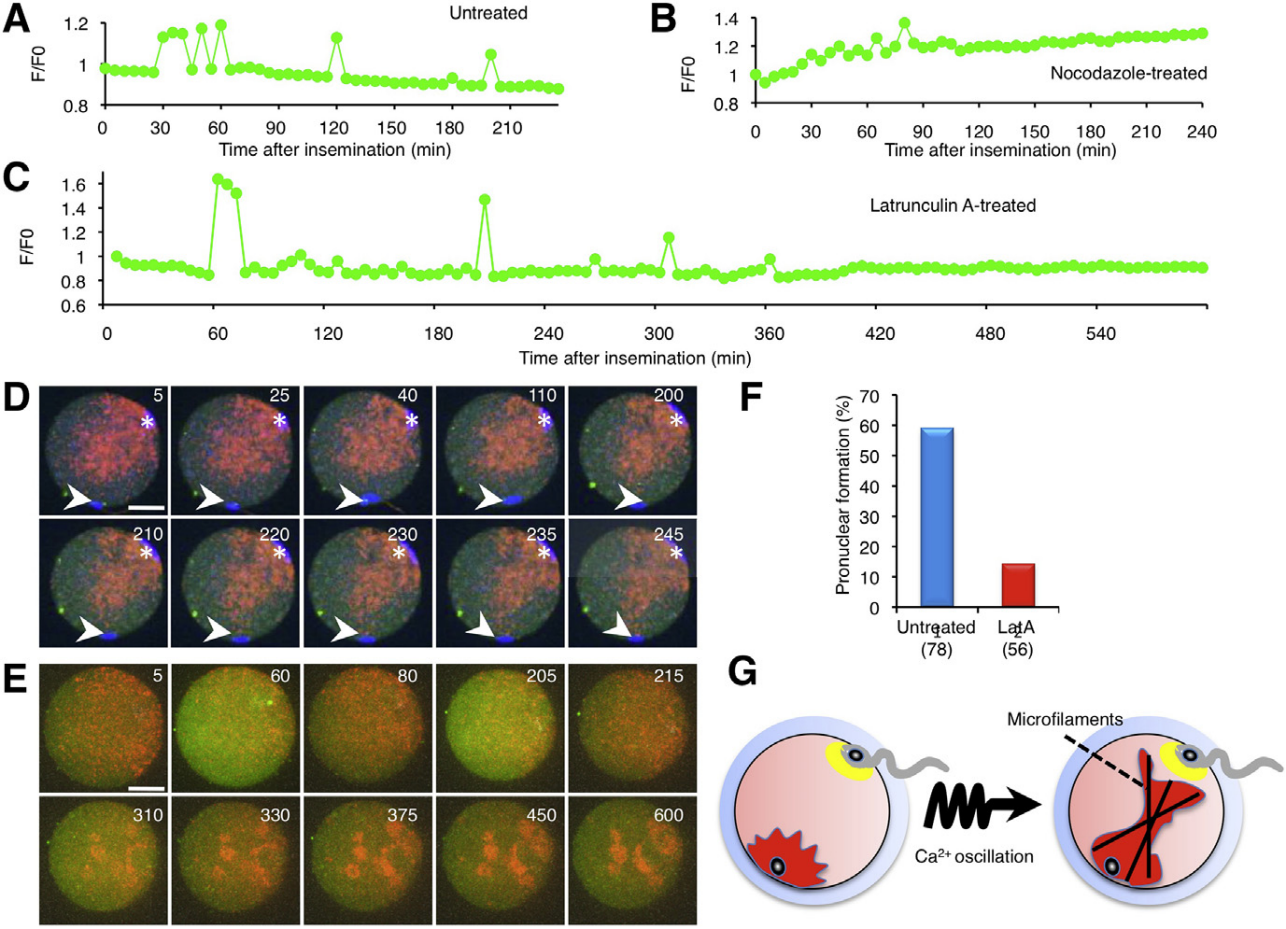
Contribution of cytoskeletons to mitochondrial movement. A, Ca^2+^ oscillation after insemination. B, Effect of nocodazole treatment on Ca^2+^ oscillation. C, Effect of latrunculin A treatment on Ca^2+^ oscillation. D, Nocodazole-treated egg. Arrowheads, fused sperm. Images (corresponding to 5, 25, 40, 110, 200, 210, 220, 230, 235, and 245 min after insemination) are shown. Scale bar, 20 μm. E, Latrunculin A-treated egg. Images (corresponding to 5, 60, 80, 205, 215, 310, 330, 375, 450, and 600 min after insemination) are shown. Scale bar, 20 μm. F, Formation of pronuclear zygotes (%). G, Schematic figure of actin-based mitochondrial chemotaxis.

To investigate the contribution of cytoskeletons to mitochondrial redistribution, we observed zona-free eggs treated with cytoskeletal disruptors (Fig. 6). We used two types of the disruptors, nocodazole that induces microtubule depolymerization [22], and latrunculin A that disrupts microfilament organization by binding to monomeric G-actin [23]. After zona-fee eggs were treated with nocodazole or latrunculin A for 10 min, they were incubated with the sperm. In nocodazole-treated eggs, the sperm fused to the egg at 20 min after insemination, and the first Ca^2+^ rise occurred. Despite no second polar body extrusion and chromosomal separation, maternal mitochondria moved and reached the sperm nucleus at 200 min after insemination (Fig. 6B and D). Ca^2+^ rises occurred 5 times from 30 to 90 min, but their peaks were small (Fig. 6B). This result suggests that mitochondrial redistribution is independent from microtubule organization.

In latrunculin A-treated eggs, the sperm fused to the egg at 65 min after insemination, and the first Ca^2+^ rise occurred. Similarly to the nocodazole-treated eggs, the second polar body extrusion was disabled in the latrunculin A-treated eggs (Fig. 6C and E). Notably, latrunculin A treatment completely inhibited mitochondrial redistribution and lead to the formation of mitochondrial aggregates (Fig. 6E). This result suggests that microfilaments regulate mitochondrial redistribution.

To investigate the effect of latrunculin A treatment on pronuclear formation, we counted the rate of pronuclear zygotes. The cumulus-intact eggs were treated with latrunculin A for 10 min and incubated with sperm. When the rate of pronuclear zygotes was counted, 14.3% of inseminated eggs developed to the pronuclear zygote in the latrunculin A-treated eggs, compared to 59% of inseminated eggs. Latrunculin A treatment had no effect on pronuclear formation in uninseminated eggs. This result indicates that microfilaments also contribute to pronuclear formation (Fig. 6F).

## 4 Discussion

We explored the relationship between mitochondrial activity and the intracellular Ca^2+^ concentration in eggs (Fig. 6G). First, we showed that sperm fusion-triggered Ca^2+^ oscillation is linked to mitochondrial activity, presumably via interactions with the ER. Second, sperm-egg fusion triggers Ca^2+^ oscillation-powered mitochondrial chemotaxis (mitotaxis) toward the fused sperm. Finally, mitochondrial redistribution depends on actin microfilament.

In many organisms, a transient intracellular Ca^2+^ rise occurs in eggs after sperm fusion and is essential for the resumption of meiosis and egg activation [24]. Ca^2+^ is released from mitochondria in sea urchins [25], and ATP molecules produced in mitochondria function in pacemaker Ca^2+^ oscillations in ascidians [26]. Our results show that mitochondria also play a crucial role in Ca^2+^ signaling as a Ca^2+^ reservoir in mice (Fig. 2B, C).

In yeasts, the multi-subunit ER-mitochondria encounter structure, which tethers the ER and mitochondria, is both spatially and functionally linked to sites of mitochondrial division [17, 18, 19]. As reported previously [26, 27], small patches of ER localize underneath the egg plasma membrane in unfertilized eggs and partially colocalize with mitochondria, as supported by our results (Fig. 2D). In addition, our results suggest that in fertilized eggs, the ER is internalized, form a large cluster, and colocalizes with mitochondria (Fig. 2D, E). In mammals, PIP2 hydrolysis generate IP3, which activates IP3 receptor-mediated Ca^2+^ release from ER [28]. Ca^2+^ flux from the ER to mitochondria is a major determinant of some mitochondrial functions [29]. Since the dysfunction of this Ca^2+^ flux causes several human diseases [30], its tight regulation is crucial for cellular homeostasis. Our results indicate that ER and mitochondria may cooperatively function in fertilized mouse eggs as well.

Microinjection of sperm extracts is known to induce Ca^2+^ oscillation, despite the absence of sperm fusion [16]. However, in our study, sperm extracts were unable to promote mitotaxis (Fig. 4C, D). The sperm structures were sustained after ultrasonication, and nucleotides were below the detectable level in the mSE (Fig. 5). These results indicate that the mSE lacks a major part of genomic DNA and RNA, implying that sperm nucleotides attract egg mitochondria after sperm fusion.

Microfilaments directly implicate in mitochondrial ATP production and distribution during oocyte maturation [31]. Furthermore, our result revealed that microfilaments regulate mitochondrial redistribution.

Compatibility between the mitochondrion and nucleus is essential for somatic cell nuclear transfer [32], and optimal component pairing is essential for efficient cellular respiration [32] and defeat of ROS [33]. Egg mitochondria are expected to have a selective advantage for paternal mitochondria. Our study improves our understanding of the molecular mechanisms underlying the matching of mitochondria with sperm nuclei after fertilization.

## Declarations

### Author contribution statement

Maki Iwai: Performed the experiments; Wrote the paper.

Yuichirou Harada: Conceived and designed the experiments; Performed the experiments; Analyzed and interpreted the data; Wrote the paper.

Rinako Miyabayashi, Woojin Kang, Akihiro Nakamura, Natsuko Kawano, Yoshitaka Miyamoto: Performed the experiments.

Mitsutoshi Yamada, Toshio Hamatani, Mami Miyado, Keiichi Yoshida, Hidekazu Saito, Mamoru Tanaka, Akihiro Umezawa: Analyzed and interpreted the data.

Kenji Miyado: Conceived and designed the experiments; Analyzed and interpreted the data; Wrote the paper.

### Funding statement

This work was supported by the Grant-in-aids for Scientific Research from the Japan Society for the Promotion of Science (No. 26670733 and No. 26293363 to K. Miyado, No. 17K16850 to K. Yoshida, No. 16KK0192 to Y. Miyamoto, and No. 18K16824 to M. Iwai).

### Competing interest statement

The authors declare no conflict of interest.

### Additional information

No additional information is available for this paper.

## Appendix A Supplementary data

**Movie 1**. Monitoring of Ca^2+^ oscillation and mitochondrial membrane potential in a fertilized egg.

Supplemental Movie 1 Supplemental Movie 1

**Movie 2**. Monitoring of Ca^2+^ oscillation and mitochondrial membrane potential in a FCCP-treated, unfertilized egg in M2 medium.

Supplemental Movie 2 Supplemental Movie 2

**Movie 3**. Monitoring of Ca^2+^ oscillation and mitochondrial membrane potential in a FCCP-treated, unfertilized egg in Ca^2+^-free M2 medium.

Supplemental Movie 3 Supplemental Movie 3

**Movie 4**. Monitoring of Ca^2+^ oscillation and mitochondrial distribution in a fertilized egg.

Supplemental Movie 4 Supplemental Movie 4

**Movie 5**. Monitoring of Ca^2+^ oscillation and mitochondrial distribution in an unfertilized egg.

Supplemental Movie 5 Supplemental Movie 5

**Movie 6**. Monitoring of Ca^2+^ oscillation and mitochondrial distribution in a mSE-injected egg.
